# Fluid Extract of Gelseminum as a Cure of Neuralgia of the Fifth Pair of Nerves

**Published:** 1876-03

**Authors:** N. B. Acheson

**Affiliations:** Youngstown, Ohio


### FLUID EXTRACT OF GELSEMINUM AS A CURE OF NEURALGIA OF THE FIFTH PAIR OF NERVES.

BY N. B. ACHESON, YOUNGSTOWN, OHIO

My attention was called to this remedy a short time ago by a practicing physician of this place who recommended it as a remedy for facial neuralgia. Since that time I have used it with much success, not only in neuralgia of the face and head, but also in cases of ordinary tooth ache complicated with inflammation of the investing membrane of the root of the tooth, and in cases of tooth ache caused by teeth in a state of ulceration. One great advantage in the use of this medicine is that it not only gives relief quickly, but does so without causing any nauseating effects as most other remedies do. Below I give four of the most important of those cases that have come under my observation and treatment.

December 28, 1875, Miss L. C., aged twenty, complained of severe neuralgic pains in left side of face and great pain in a tooth in left side, lower jaw. Examination revealed the root of the second left inferior bicuspid to be in a state of ulceration, but as she was too timid to have it extracted, I gave her one dose of five drops of gelseminum in a teaspoonful of water and in less than fifteen minutes she was entirely free from pain until the next day, when, by an accident the neuralgia was started afresh when I administered a similar dose of the same medicine and she was soon relieved and has had no return of the pain since.

December 30, 1875, Miss T. M., aged eighteen, suffering from severe neuralgia in right side of face. Two doses of gelseminum administered three hours apart, completely cured her. Her trouble was caused by an ulcerated first right superior molar which she wished to save.

December 27, 1875, Miss L. B., aged 25, had the second right superior bicuspid, root and crown filled by me, as the filling was large and difficult of access, she of course suffered much during the operation. I applied a counter irritant to the sensitive part with but little success. December 28, she complained of severe neuralgic pains starting from that tooth and that she had no sleep during the night. One dose of five drops of gelseminum completely relieved her.

January 5, 1876, Mrs. S. S., complained of terrible neuralgic pains over the right side of head and face, and especially in right side of lower jaw where her teeth were all extracted. She had those teeth extracted over a year ago, then to get rid of neuralgia, but has been bothered with it, with more or less severity, ever since, until I gave her one dose of five drops of gelseminum which relieved her entirely.

In as much as this medicine has not been put forward among dentists as a remedy for neuralgia, and as every dentist will be interested in using something that will be of so much service in alleviating pain, I hope that it will be more generally used and that others will add their experience for the benefit of us all. I would say in connection with this that I have had no case in which I have not been able to very materially relieve if not absolutely cure it.

